# Supplementary material for: Enterocytes rely on purine biosynthesis/salvage pathway to facilitate dietary fat absorption
Source: Nat Commun. 2026 Mar 13;17:3888. doi: 10.1038/s41467-026-70332-3 (PMC13125253; doi:10.1038/s41467-026-70332-3)
Supplement: Supplementary file 1 — Supplementary Information [file 41467_2026_70332_MOESM1_ESM.pdf]

Supplementary Information for

Enterocytes Rely On Purine Biosynthesis/Salvage Pathway To Facilitate Dietary Fat Absorption

Yu Wang[1,*], Li Chen[2,3], Yingze Ma[1], Mingqi Zhou[4], Aleksander Geske[5], Marcus Seldin[4], Jiangjiang Zhu[2,3], Alexander M Zak[1], Xinzhong Dong[5,6], Robert N Cole[7], and Svetlana Lutsenko[1,*]

Affiliations:

[1] Department of Physiology, Pharmacology & Therapeutics, Johns Hopkins University School of Medicine, Baltimore, Maryland 21205, USA;

[2] Human Nutrition Program, Department of Human Sciences, The Ohio State University, Columbus, OH 43210, USA;

[3] James Comprehensive Cancer Center, The Ohio State University, Columbus, OH 43210, USA;

[4] Department of Biological Chemistry, University of California School of Medicine, Irvine, CA 92697, USA;

[5] Solomon H. Snyder Department of Neuroscience, Johns Hopkins University School of Medicine, Baltimore, MD 21205, USA.

[6] Howard Hughes Medical Institute, Chevy Chase, MD 20815, USA.

[7] Department of Biological Chemistry, Johns Hopkins University School of Medicine, Baltimore, Maryland, 21205, USA

* Correspondence should be addressed to: Svetlana Lutsenko, Ph.D. (lutsenko@jhmi.edu) or Yu Wang, Ph.D. (ywang391@jh.edu). Department of Physiology, Pharmacology & Therapeutics, School of Medicine, Johns Hopkins University, Hunterian Building Room 203, 725 North Wolfe Street, Baltimore, Maryland, 21205, USA. Phone: +1(410)-614-4661.

PDF file includes:

- Supplementary Figure 1 to 15
- Supplementary Table 1
- Supplementary Methods

Supplementary Figure 1

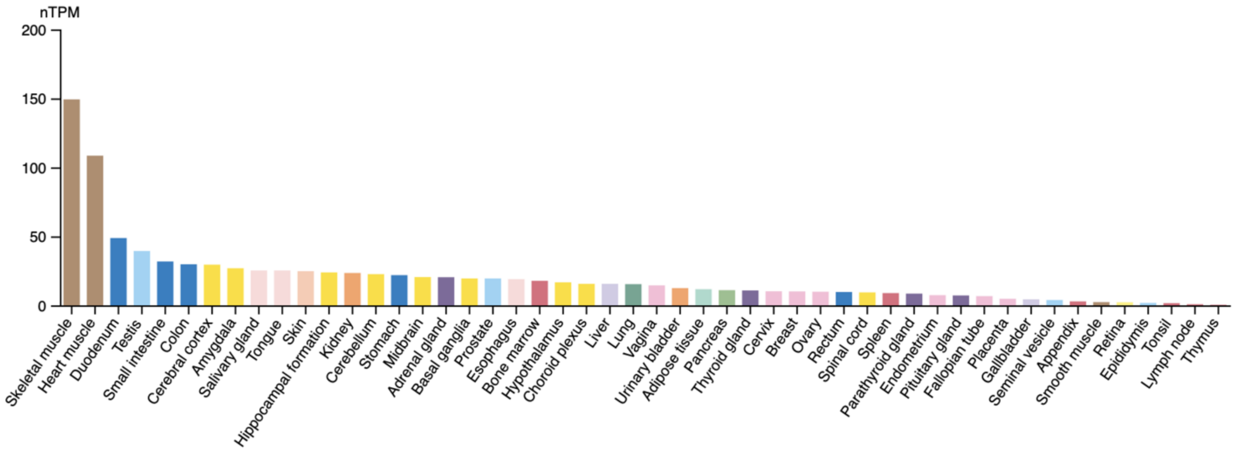

Supplementary Figure 1. ANKRD9 mRNA levels in different tissues

ANKRD9 mRNA expression in tissues from the Consensus dataset of the Human Protein Atlas (https://www.proteinatlas.org/ENSG00000156381-ANKRD9/tissue).

Supplementary Figure 2

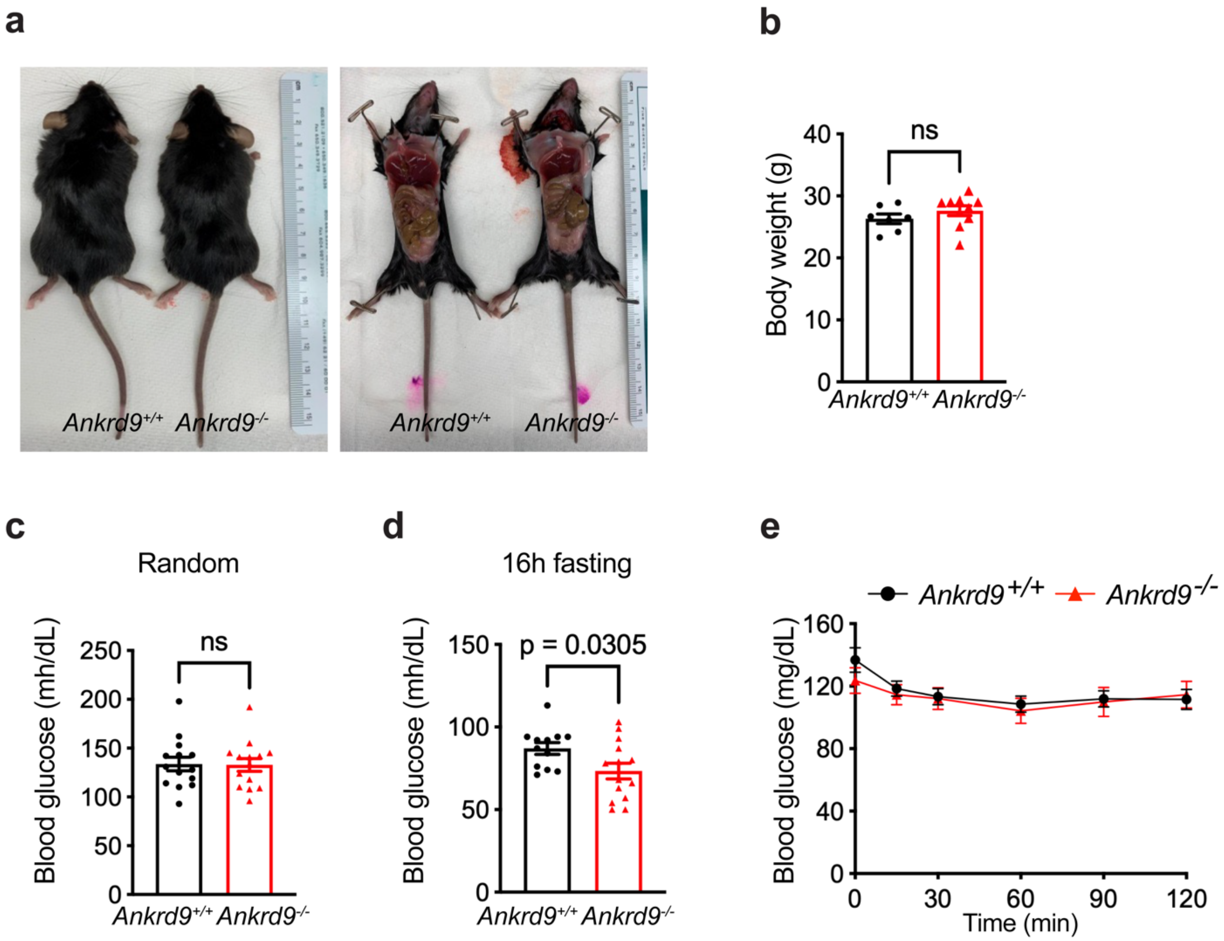

Supplementary Figure 2. Basic characteristics of *Ankrd9$^{-/-}$* mice

(a) Images of control *Ankrd9$^{+/+}$* and *Ankrd9$^{-/-}$* male mice at 14 weeks show similar size (left) and fat depots (right). (b) At 14 weeks old *Ankrd9$^{+/+}$* and *Ankrd9$^{-/-}$* mice have the same body weight; n= 7~10 mice per group, please refer to the Source Data File. (c-d) Blood glucose levels in 14 weeks old *Ankrd9$^{+/+}$* and *Ankrd9$^{-/-}$* mice, n=12~14 mice per group, please refer to the Source Data File. (e) Insulin tolerance test (1 unit per kg body weight) following 4h fasting show similar sensitivity to insulin; n=7~10 mice per group, please refer to the Source Data File; (b-e) p-values as indicated by a two-tailed unpaired t-test; Data are shown as the mean ± SEM.

Supplementary Figure 3

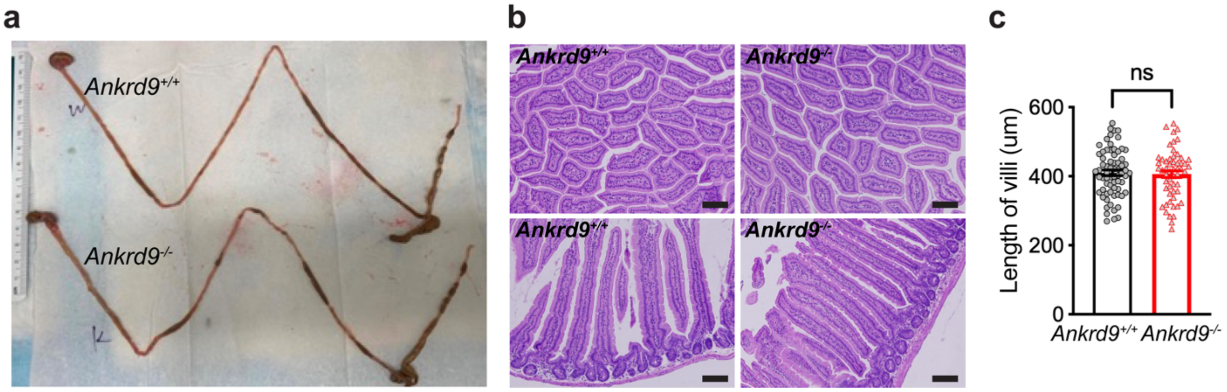

Supplementary Figure 3. Inactivation of Ankrd9 does not affect gross intestinal morphology

(a) The whole jejunum of *Ankrd9*$^{+/+}$ and *Ankrd9*$^{-/-}$ male mice at 14 weeks. (b) The H&E staining of tissue sections from *Ankrd9*$^{+/+}$ and *Ankrd9*$^{-/-}$ jejunum show normal architecture of crypts and villi; n= 4 *Ankrd9*$^{+/+}$ and 5 *Ankrd9*$^{-/-}$ mice per group. (c) Quantitation of jejunal villi length; n = 68 villi from 4 *Ankrd9*$^{+/+}$ mice and 55 villi from 5 *Ankrd9*$^{-/-}$ mice, please refer to the Source Data File; p-values as indicated by a two-tailed unpaired t-test; Data are shown as the mean ± SEM.

Supplementary Figure 4

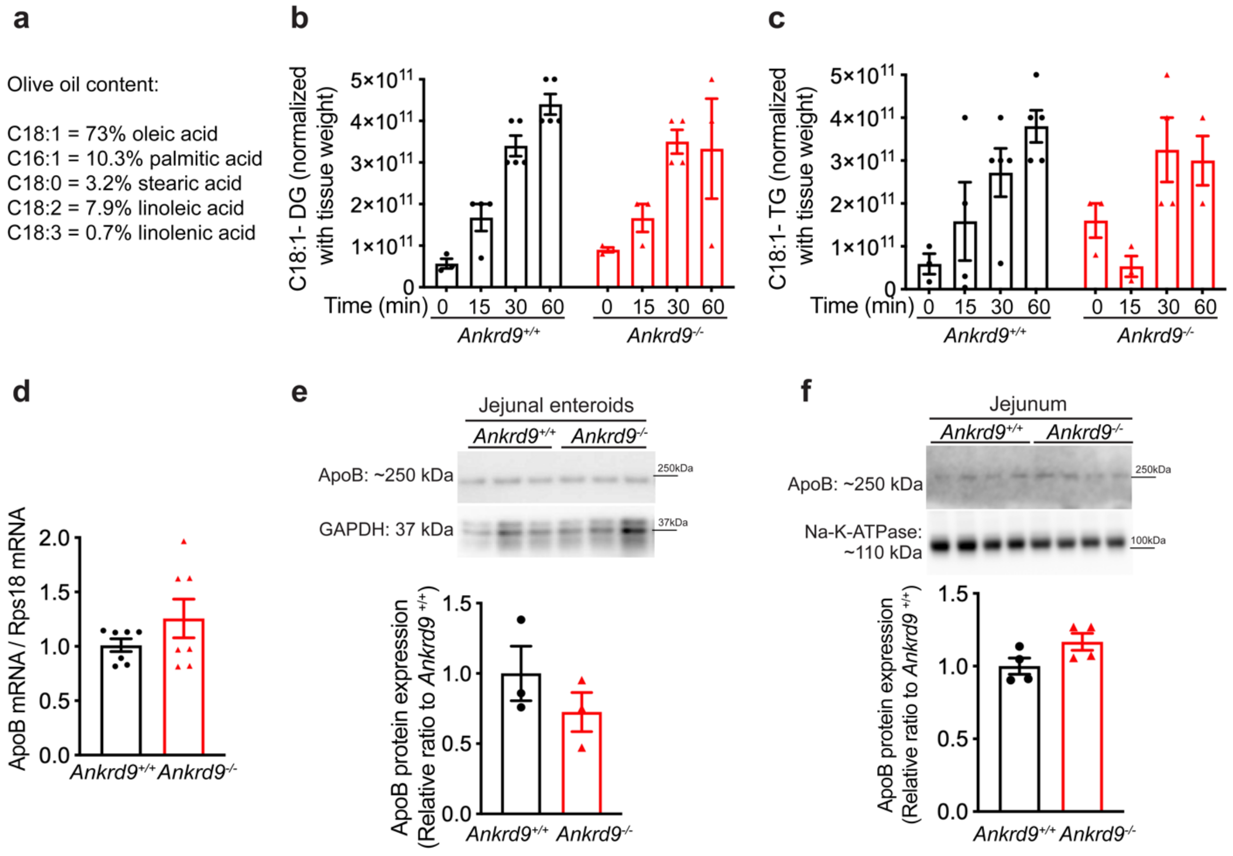

Supplementary Figure 4. The ApoB expression is not significantly changed in *Ankrd9$^{-/-}$* enteroids

(a) Olive oil components. (b) Jejunal DG (C18:1) and (c) TG (C18:1) level during oil treatment; The result was normalized to tissue weight; n = 3~5 mice per group at each time point, please refer to the Source Data File. (d) The ApoB mRNA expression in *Ankrd9$^{+/+}$* and *Ankrd9$^{-/-}$* jejunal enteroids analyzed by qPCR; n = 7 individual samples per group, please refer to the Source Data File. (e) Western blot analysis of enteroids homogenates (upper) and densitometry (lower) show unaltered protein abundance of ApoB in *Ankrd9$^{+/+}$* and *Ankrd9$^{-/-}$* enteroids; n = 3 individual samples per group, please refer to the Source Data File. (f) Western blot analysis of jejunal homogenates (upper) and densitometry (lower) show unaltered protein abundance of ApoB in *Ankrd9$^{+/+}$* and *Ankrd9$^{-/-}$* jejunum; n = 4 mice per group, please refer to the Source Data File**.** (b-f) Data are shown as the mean ± SEM. (e-f) Uncropped images of blots are shown in the Source Data File; Data represent three independent experiments.

Supplementary Figure 5

a

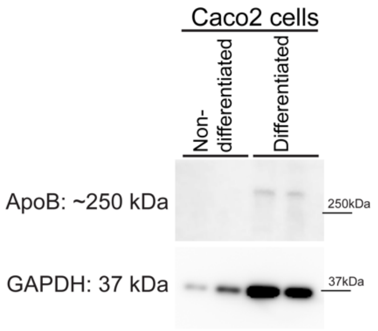

b

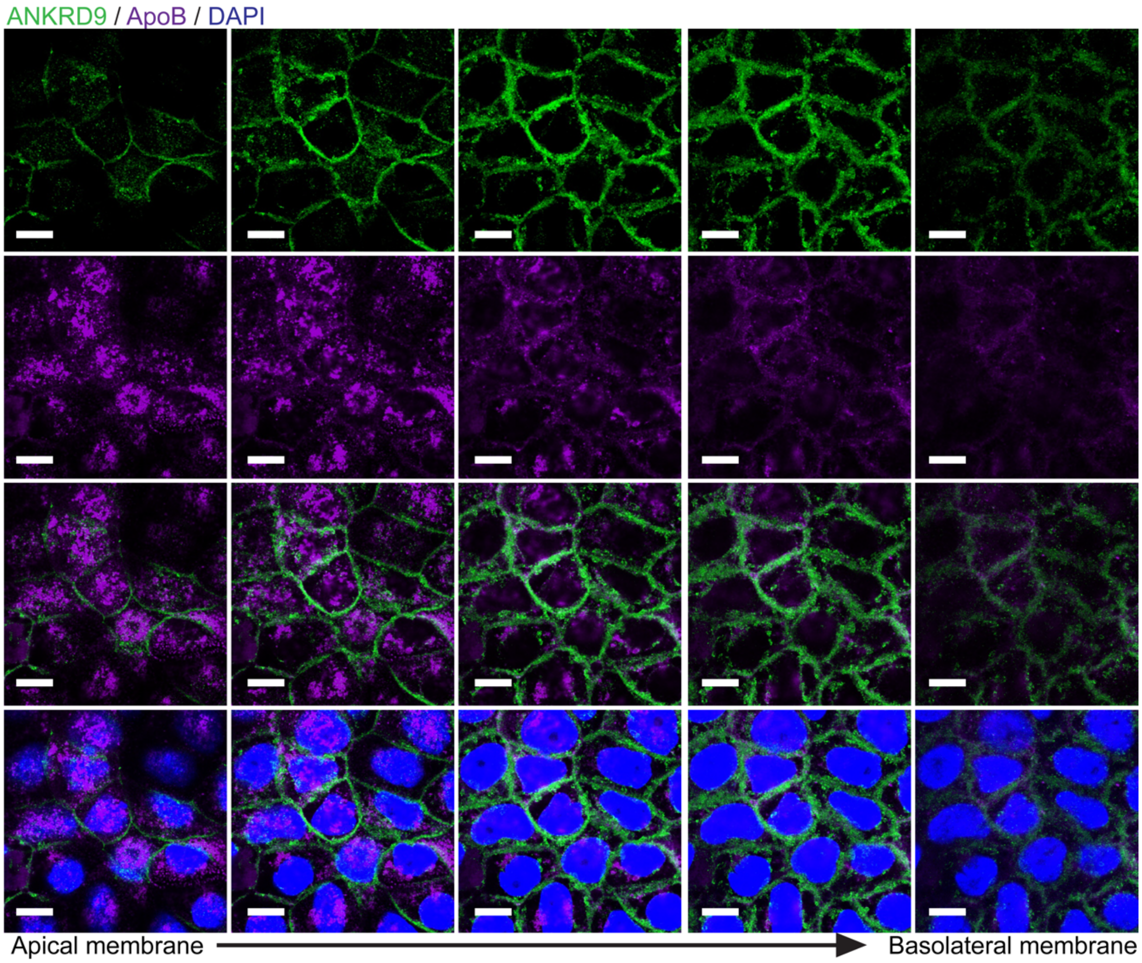

Supplementary Figure 5. ANKRD9 does not interact with ApoB

(a) Protein abundance of ApoB in non-differentiated and differentiated Caco2 cells. Uncropped images of blots are shown in the Source Data File. (b) Immunohistochemical staining of endogenous ANKRD9 (green) and ApoB (purple) in differentiated Caco2 cells; images are shown from apical to basolateral membrane; Scale bar: 10 um; Data represent three independent experiments.

Supplementary Figure 6

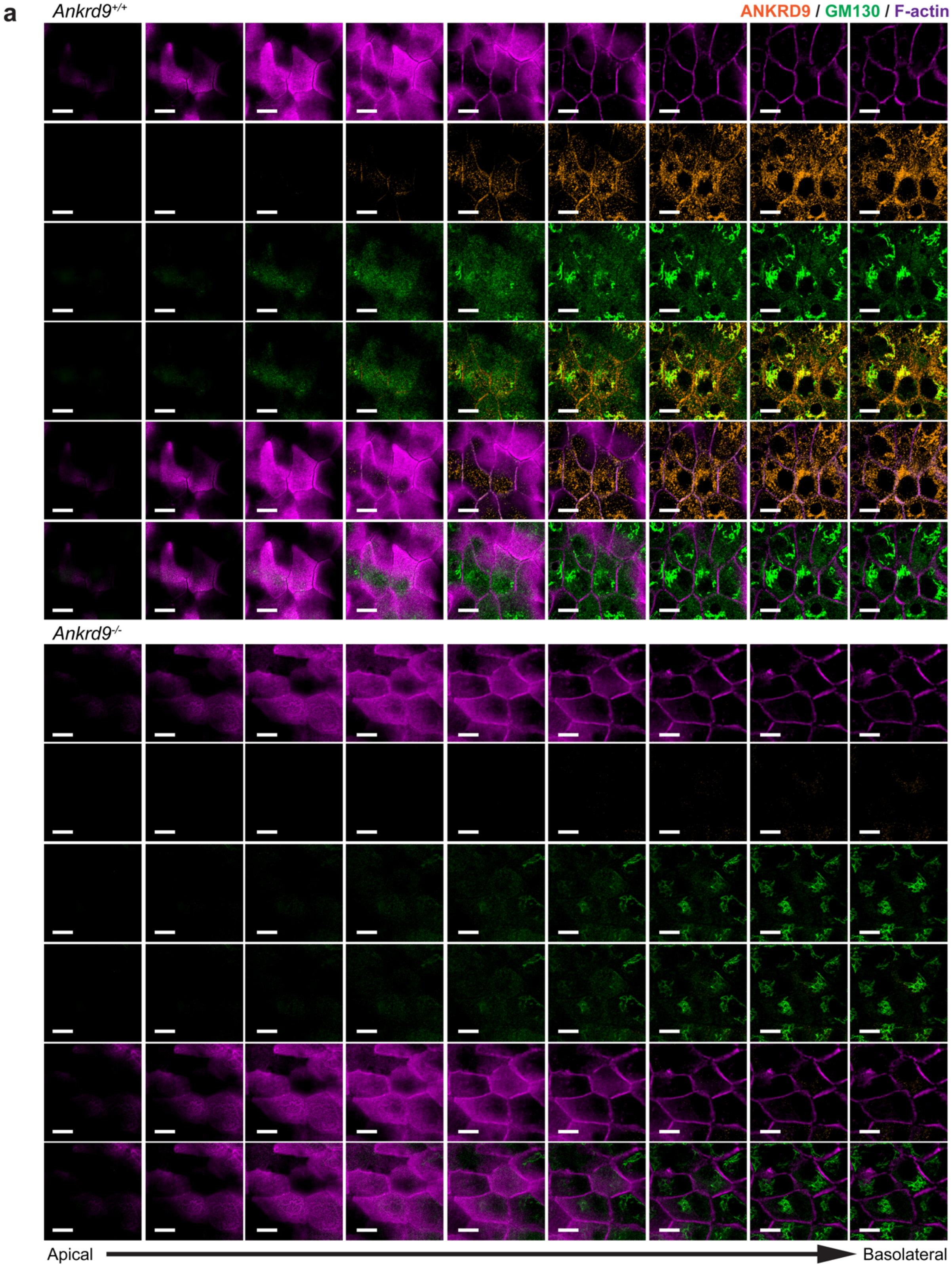

Supplementary Figure 6

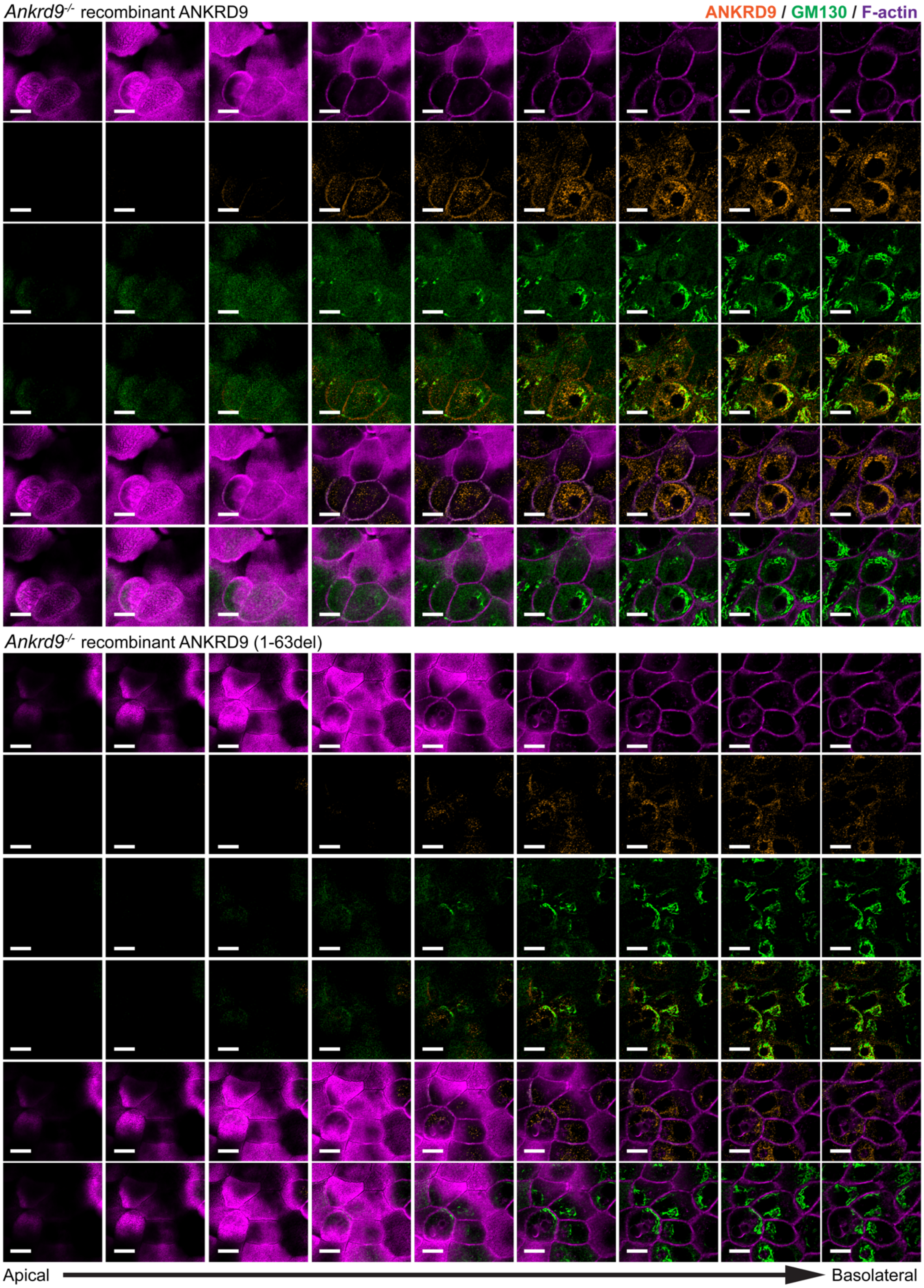

Supplementary Figure 6

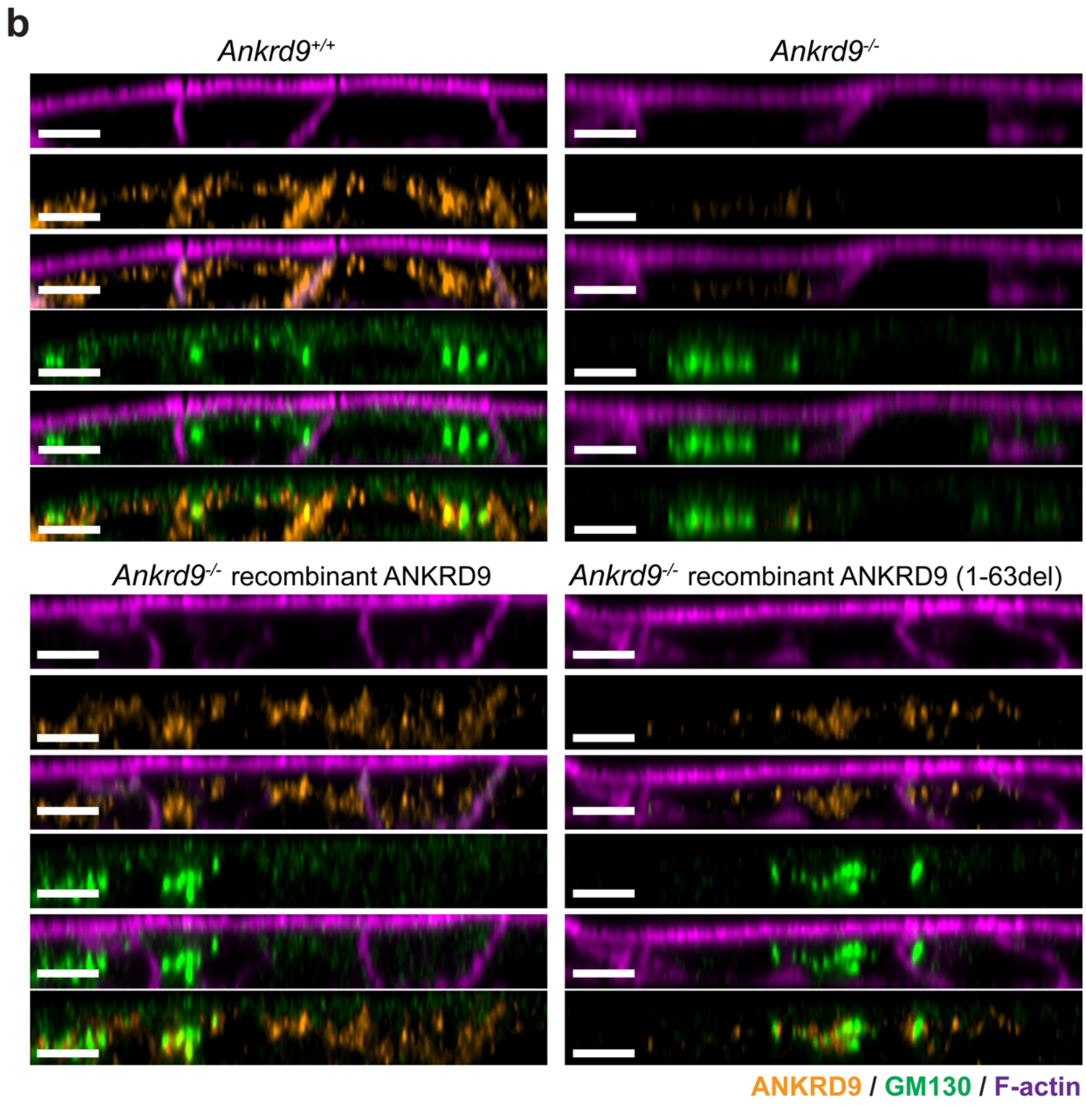

Supplementary Figure 6. The spatial distribution of ANKRD9 along the apical-basolateral axis in differentiated polarized mouse enteroids

The full panel XY (a) and XZ (b) images of immunostaining of ANKRD9 (orange), F-actin (purple), and GM130 (green) and their colocalization in the differentiated enteroids monolayer. The following enteroids are shown: *Ankrd9*$^{+/+}$; *Ankrd9*$^{-/-}$; *Ankrd9*$^{-/-}$ expressing recombinant full-length ANKRD9; and *Ankrd9*$^{-/-}$ enteroids expressing ANKRD9 (1-63del) variant. (a) Scale bar: 10 um. (b) Scale bar: 5 um. (a-b) All images were taken and processed under the same experimental conditions; Data represents three independent experiments.

Supplementary Figure 7

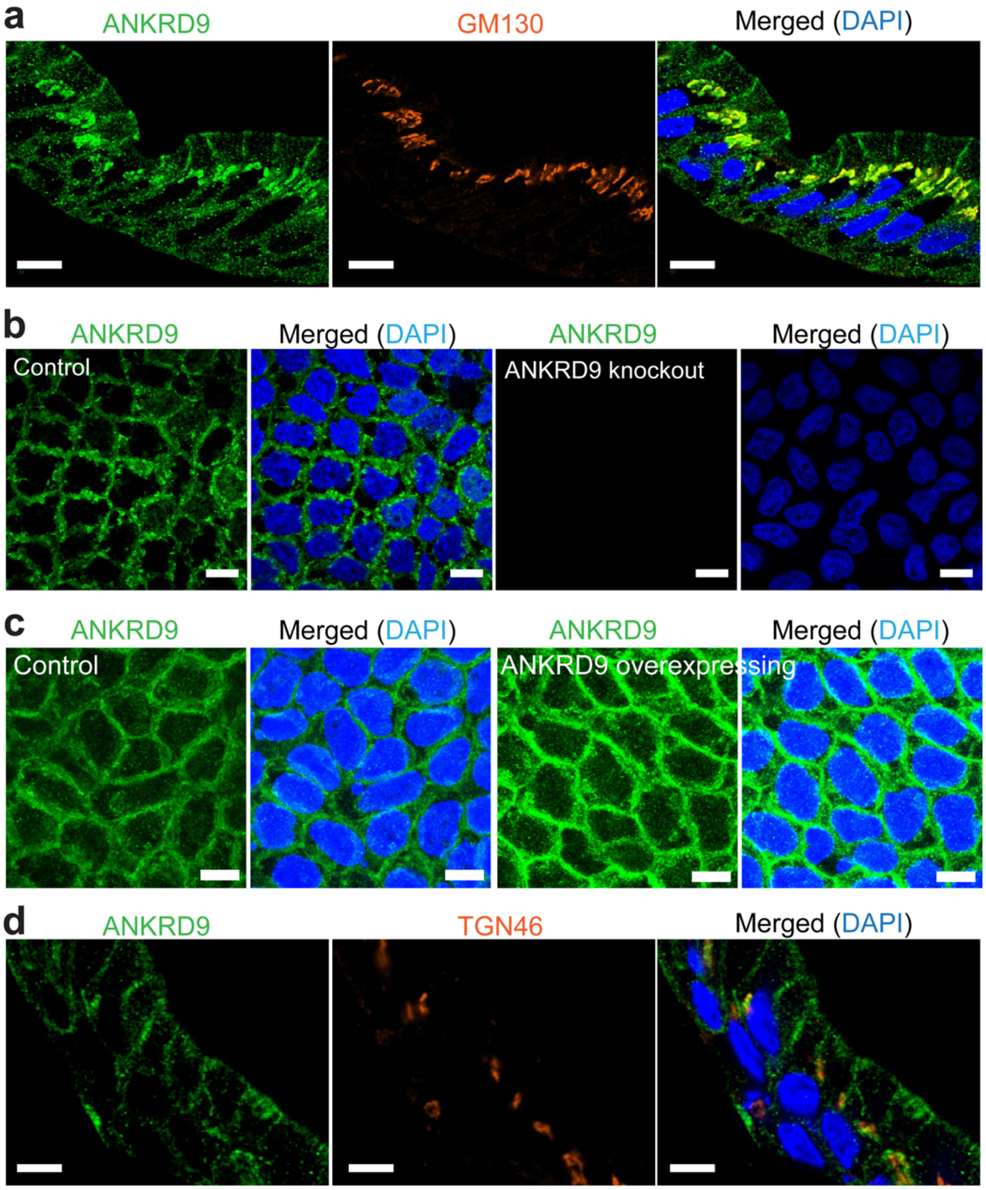

Supplementary Figure 7. The localization of endogenous ANKRD9 in enterocytes

(a) Immunostaining of ANKRD9 (green) and GM130 (orange) in differentiated 3D human jejunal enteroids. (b) Immunohistochemical staining of ANKRD9 (green) in differentiated control and ANKRD9 knockout (Cas9-ANKRD9) Caco2 cells confirms ANKRD9 deletion and the specificity of immunostaining. (c) Comparison of patterns for endogenous ANKRD9 (green) and recombinants in differentiated Caco2 cells. (d) Immunohistochemical staining of ANKRD9 (green) and TGN46 (orange) in differentiated 3D human jejunal enteroids. (a-d) Scale bar: 10 um; Data represent three independent experiments.

Supplementary Figure 8

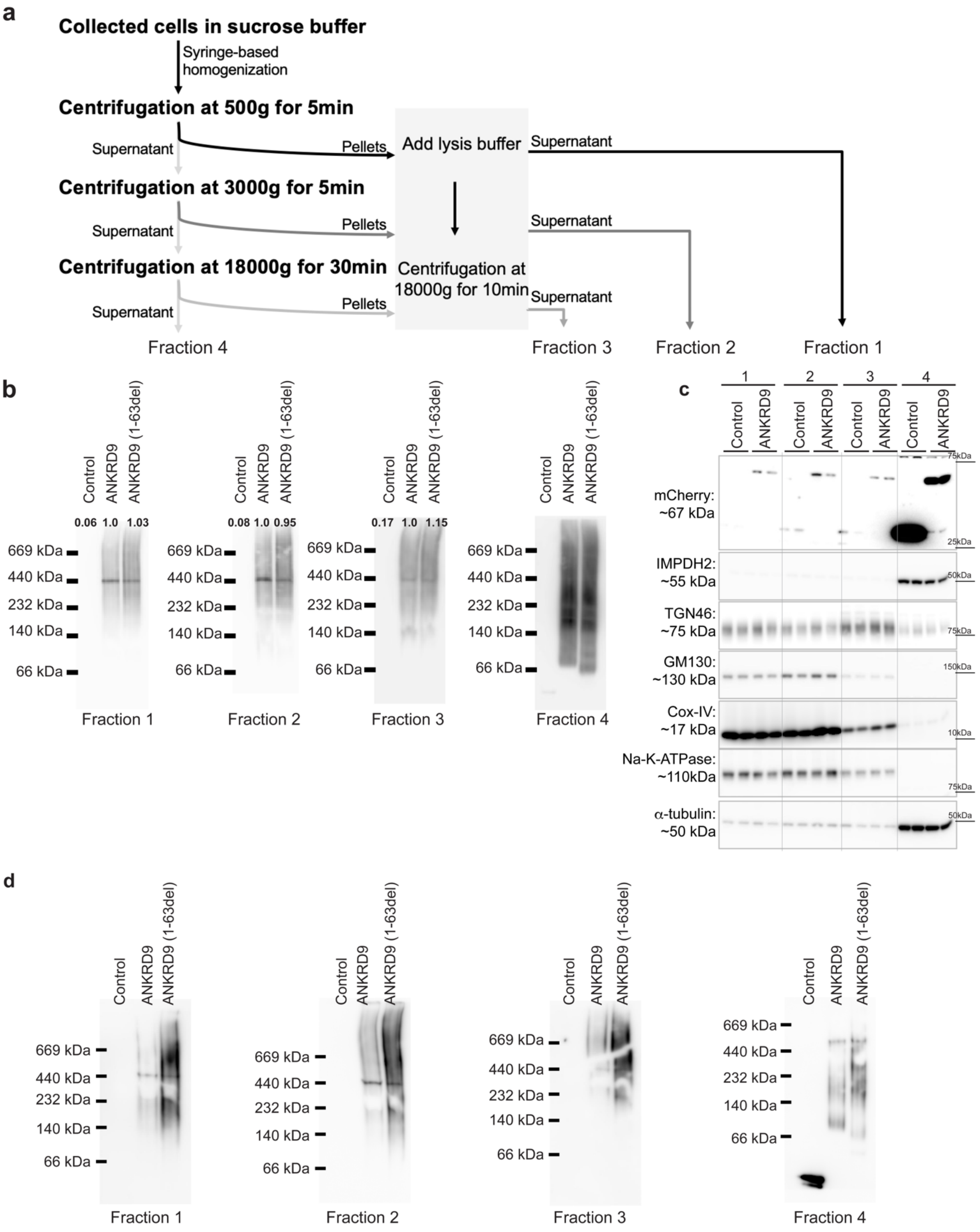

Supplementary Figure 8. Ankrd9 forms large membrane associated protein complexes near cis-Golgi

(a) Schematic of cell fractionation. (b) Western blot analysis using anti-mCherry antibody of protein complexes formed by the recombinant mCherry-ANKRD9 or mCherry ANKRD9 (1-63del) variant expressed in HEK293 cells. Fractions were produced using differential centrifugation and analyzed by Blue native gels. Western blotting and immunodetection were done using anti-mCherry antibody. Fractions 1-3 contain membrane proteins and Fraction 4 - soluble proteins. (c) Distribution of Flag-ANKRD9-mCherry and organelle markers in cytosol (mCherry, IMPDH2, α-tubulin) and membrane-containing fractions, such as Golgi (GM130), mitochondria (Cox-IV), and plasma membranes (Na/K-ATPase); Uncropped images of blots are shown in the Source Data File. (d) Blue native gel separation and Western blot analysis of fractions generated from differentiated Caco2 cells expressing ANKRD9-mCherry or ANKRD9(1-63del)-mCherry; the ANKRD9-containing complexes were detected with anti-mCherry antibody. (b-d) Data represent three independent experiments.

Supplementary Figure 9

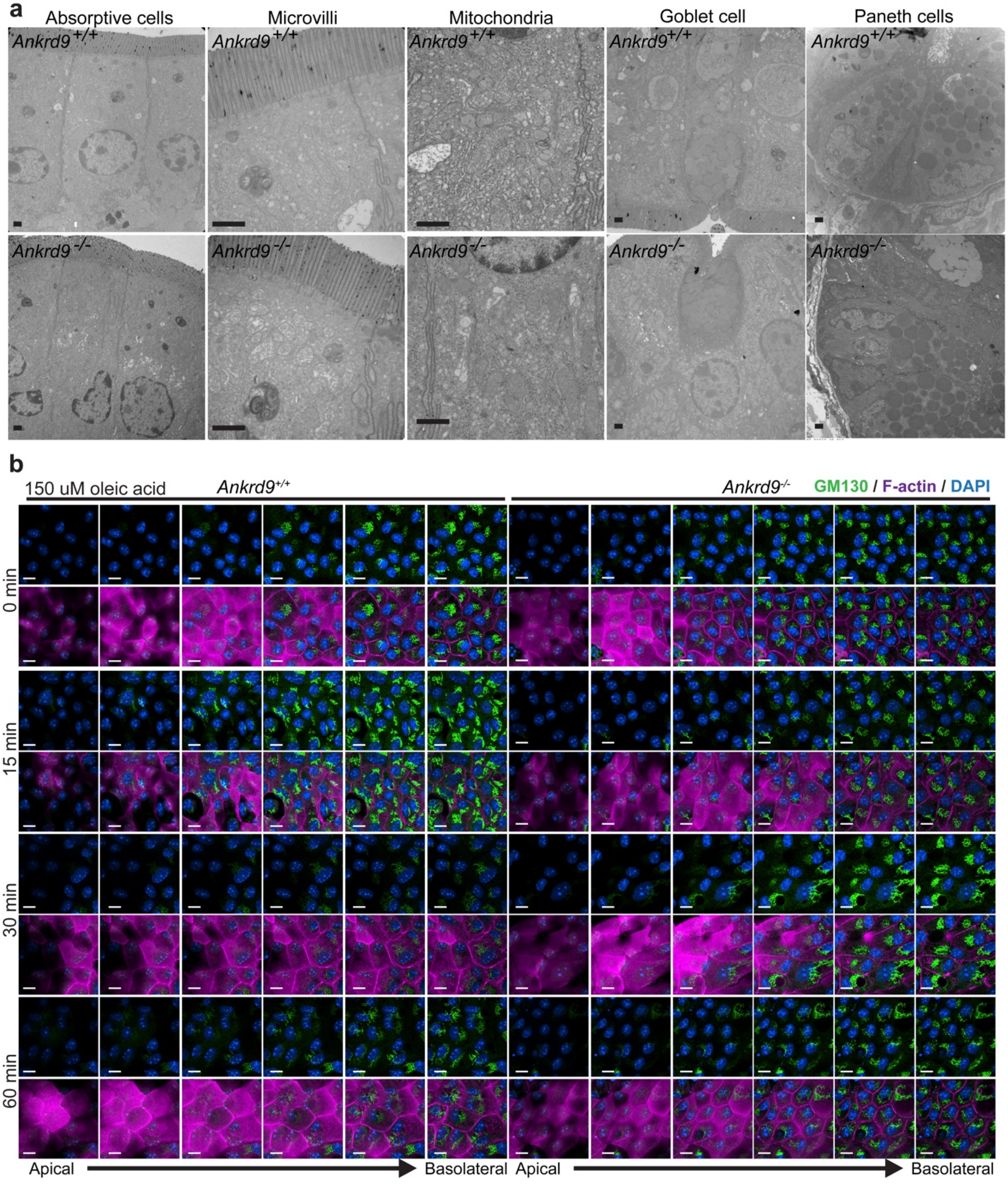

Supplementary Figure 9. Inactivation of ANKRD9 does not alter the morphology of jejunal cells

(a) The EM images of microvilli and mitochondria in absorptive cells, goblet cells, and Paneth cells from *Ankrd9*$^{+/+}$ and *Ankrd9*$^{-/-}$ jejunum; n = 3 mice per group; Scale bar: 500 nm. (b) Immunohistochemical staining of GM130 (green) in *Ankrd9*$^{+/+}$ and *Ankrd9*$^{-/-}$ jejunum during fat treatment show Golgi expansion towards apical membrane (marked by F-actin (purple)) at 15 min, whereas this response is delayed (at 30 min) and less pronounced in *Ankrd9*$^{-/-}$ jejunum. Scale bar: 10 um; Data represents three independent experiments.

Supplementary Figure 10

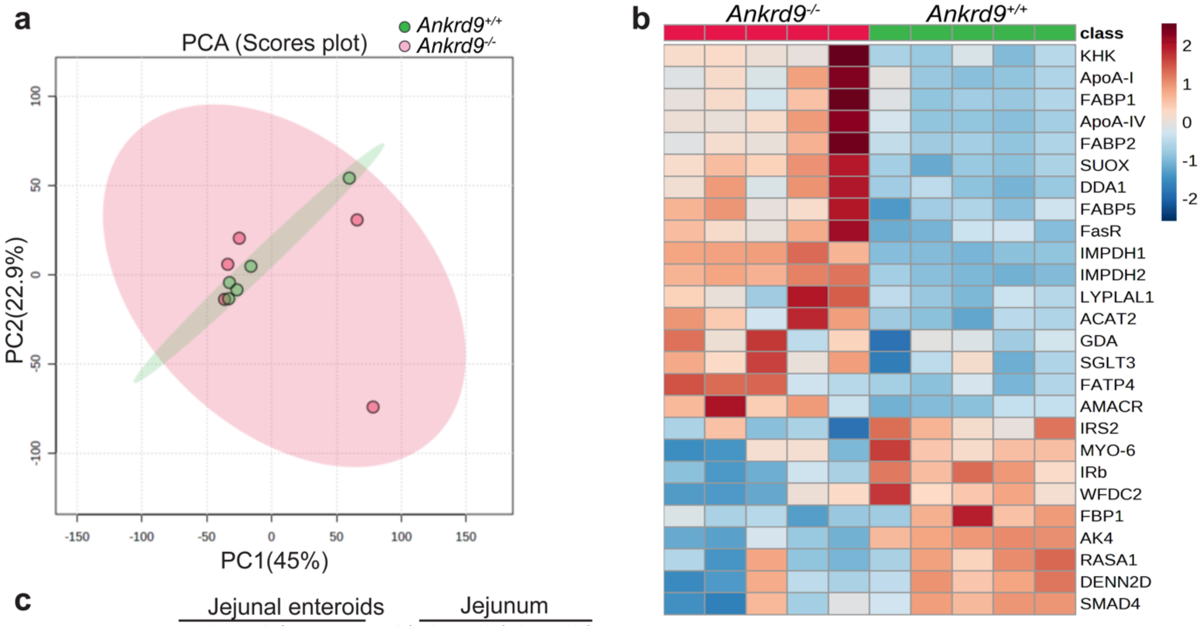

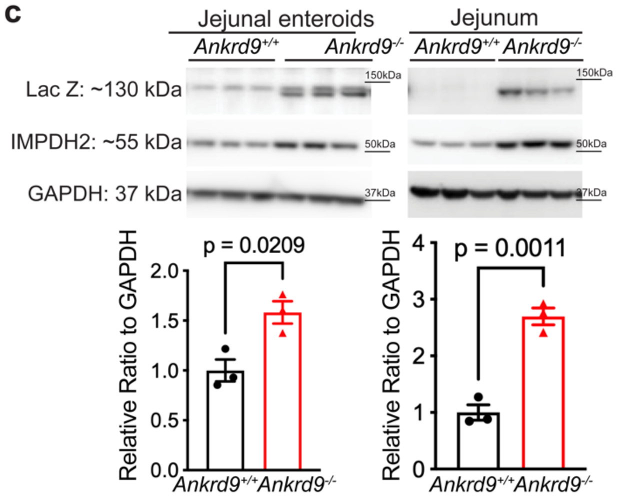

d

	Ankrd9 +/+	*Ankrd9* -/-	P-Value
**ATP**	36.049 ± 2.644	24.831 ± 3.389	0.0229
**ADP**	3.796 ± 0.119	7.898 ± 1.029	0.0052
**AMP**	0.763 ± 0.064	0.673 ± 0.074	0.3738
**GTP**	8.132 ± 0.469	5.093 ± 0.722	0.0045
**GDP**	1.742 ± 0.072	3.337 ± 0.522	0.0183
**GMP**	0.215 ± 0.012	0.189 ± 0.022	0.3217

Nucleotides concentration (umol /g protein)

Supplementary Figure 10. Ankrd9 inactivation is associated with an altered abundance of proteins involved in nucleotide balance and lipid transport

(a) The PCA plot showing similarity of jejunal enteroids proteome from *Ankrd9*$^{+/+}$ and *Ankrd9*$^{-/-}$ mice. (b) Heatmap showing significantly changed proteins in the jejunal enteroids of *Ankrd9*$^{-/-}$ mice. (c) Protein expression of IMPDH2 in jejunal enteroids and in jejunum tissue from *Ankrd9*$^{+/+}$ and *Ankrd9*$^{-/-}$ mice analyzed by western blot and densitometric analysis of IMPDH2, respectively; Uncropped images of blots are shown in the Source Data File; n=3 individual samples per group, each lane represents an individual mouse sample, please refer to the Source Data File; p-values as indicated by a two-tailed unpaired t-test; Data are shown as the mean ± SEM. The data represent three independent experiments. (d) Table of nucleotides concentration in jejunal enteroids of *Ankrd9*$^{+/+}$ and *Ankrd9*$^{-/-}$ mice, results are normalized with protein amount; n=6~8 individual samples per group, please refer to the Source Data File. p-values as indicated by a two-tailed unpaired t-test; Data are shown as the mean ± SEM.

Supplementary Figure 11

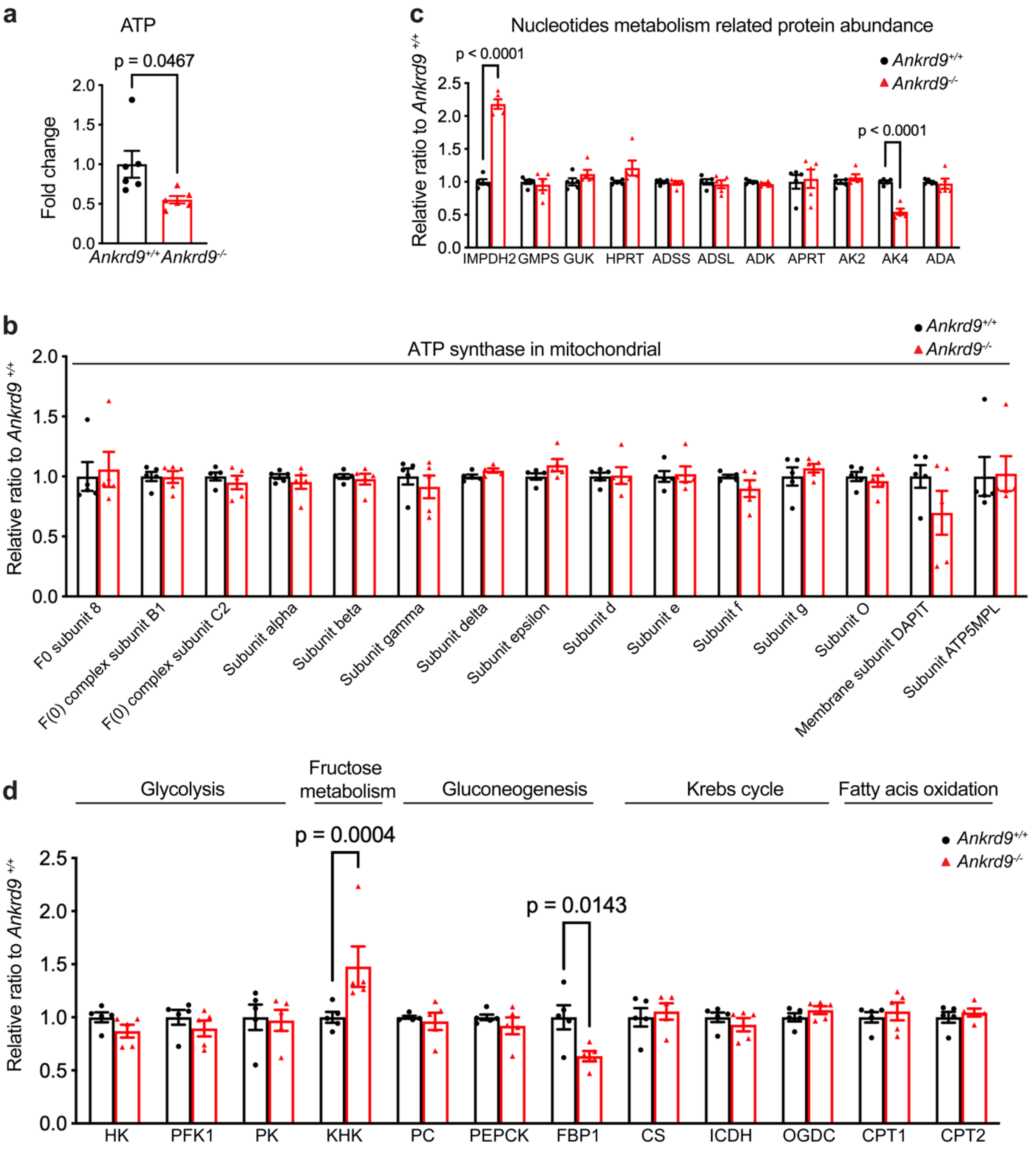

Supplementary Figure 11. Ankrd9 deletion does not affect mitochondrial respiration or glycolysis

(a) ATP levels in jejunal enteroids from *Ankrd9*$^{+/+}$ and *Ankrd9*$^{-/-}$ mice determined by luciferase-based assay. n=6 individual samples per group, and three independent experiments were performed; please refer to the Source Data File; p-values as indicated by a two-tailed unpaired t-test; Data are shown as the mean ± SEM. (b) Abundance of ATP synthase protein components in mitochondria in control and *Ankrd9*$^{-/-}$ enteroids, (c) the protein abundance of enzymes in the purine biosynthesis pathways in control and *Ankrd9*$^{-/-}$ enteroids, and (d) the abundances of enzymes associated with glycolysis, gluconeogenesis, Krebs cycle and fatty acid oxidation in *Ankrd9*$^{+/+}$ and *Ankrd9*$^{-/-}$ enteroids measured by mass-spectrometry. (b-d) n=5 individual samples per group, please refer to the Source Data File; p-values as indicated by two-way ANOVA with Šídák test; Data are shown as the mean ± SEM. GMPS: guanine monophosphate synthetase; GUK: guanylate kinase; HPRT: hypoxanthine-guanine phosphoribosyltransferase; ADSS: adenylosuccinate synthase; ADSL: adenylosuccinate lyase; ADK: adenosine kinase; APRT: adenine phosphoribosyl transferase; AK: adenylate kinase; ADA: adenosine deaminase; (d)*$p<0.05$; HK: hexokinase; PFK1: phosphohexose isomerase; PK: pyruvate kinase; KHK: ketohexokinase; PC: Pyruvate carboxylase; PEPCK: Phosphoenolpyruvate carboxykinase; FBP1: phosphohexose isomerase; CS: citrate synthase; ICDH: isocitrate dehydrogenase; OGDC: a-ketoglutarate dehydrogenase; CPT1: Carnitine palmitoyl transferase 1; CPT2: Carnitine palmitoyl transferase 2.

Supplementary Figure 12

a

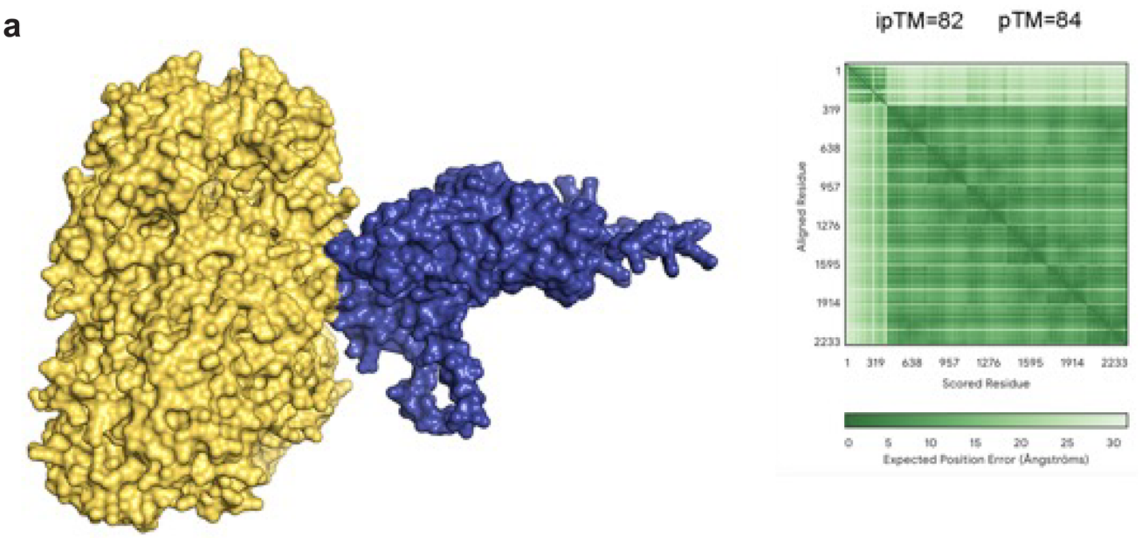

b

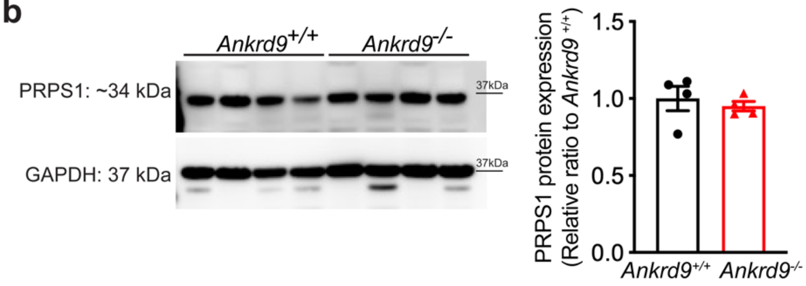

c

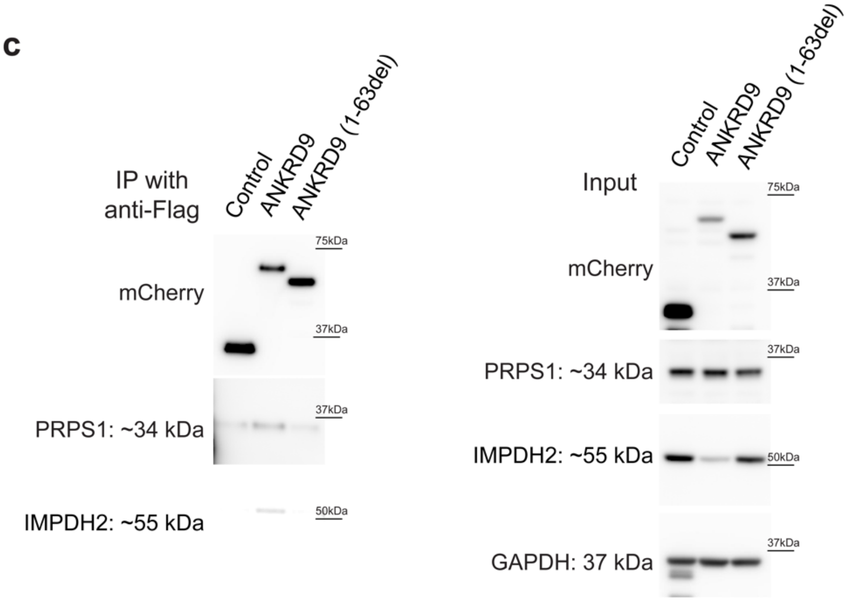

Supplementary Figure 12. ANKRD9 does not affect the PRPS1 abundance but may form a protein complex

AlphaFold predicts complex formation between an active (hexameric) form of PRPS1 (yellow) and ANKRD9 (blue). The ipTM score of 82 is indicative of likely interactions. (b) Protein expression of PRPS1 in jejunum tissues homogenates from *Ankrd9*$^{+/+}$ and *Ankrd9*$^{-/-}$ mice was analyzed by Western blot and quantified by densitometry; n=4 mice per group, each lane represents an individual mouse sample, please refer to the Source Data File; Data are shown as the mean ± SEM. (c) Caco-2 cells were transfected with full-length ANKRD9-Cherry or ANKRD9 (1-63del)-Cherry or Cherry alone (as a control), immunoprecipitated using anti-Flag magnetic beads, and the presence of PRPS1 and IMPDH2 in co-IP was detected using respective antibodies; The data represent three independent experiments. (b-c) Uncropped images of blots are shown in the Source Data File.

Supplementary Figure 13

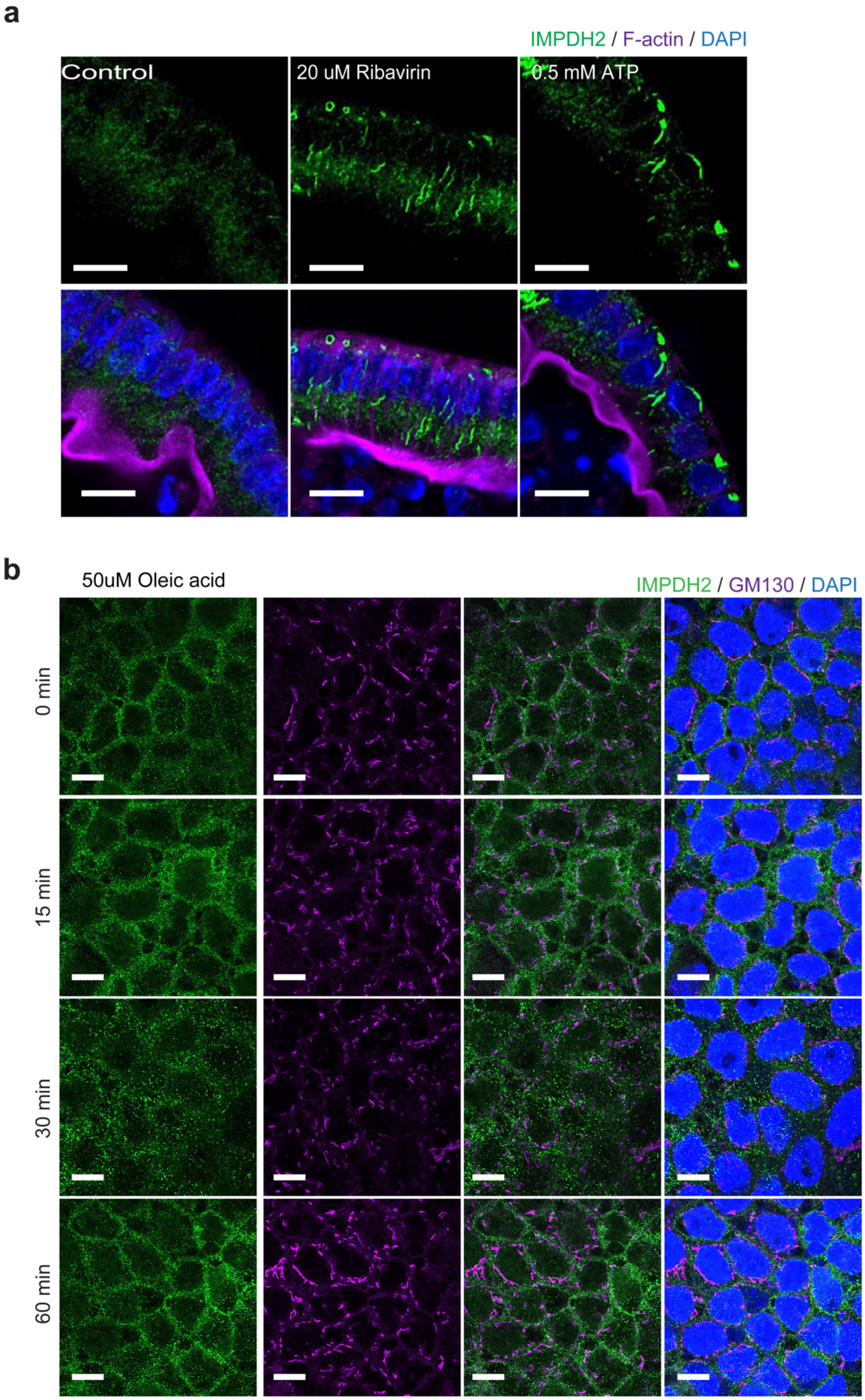

Supplementary Figure 13. ATP levels increase during lipid processing

(a) IMPDH2 (green) forms filaments in jejunal *Ankrd9*$^{+/+}$ and *Ankrd9*$^{-/-}$ enteroids in response to treatment with inhibitor 20 uM ribavirin (increase in ATP/GTP ratio) or 0.5 mM ATP for 4h. (b) Lipid induced change in IMPDH2 (green) pattern in differentiated Caco2 cells following 50 uM oleic acid treatment for 0, 15, 30 and 60 min. (a-b) Scale bar: 10 um; The data represent three independent experiments.

Supplementary Figure 14

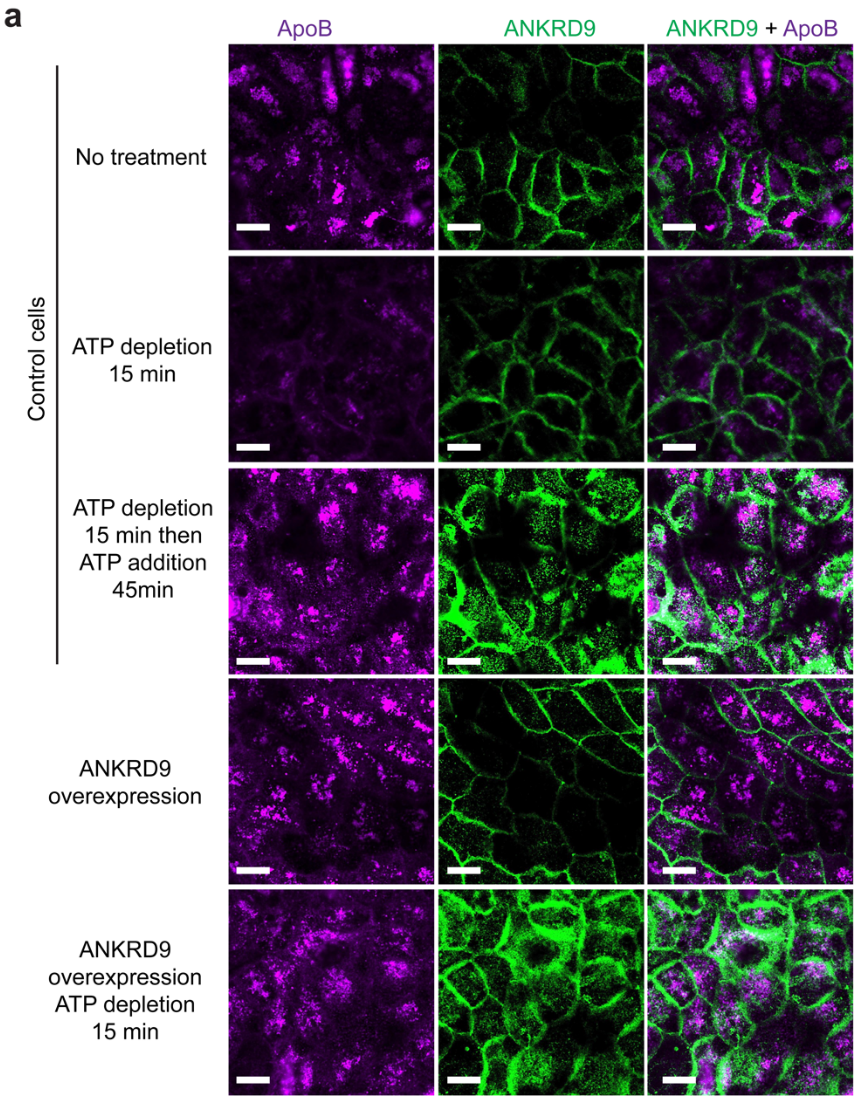

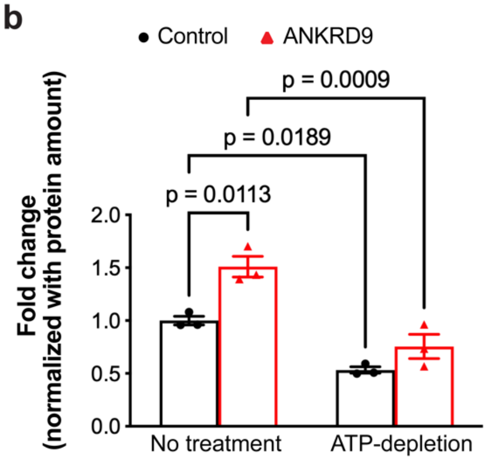

Supplementary Figure 14. Recombinant ANKRD9 rescues ApoB localization at the apical membrane

(a) Immunohistochemical staining of ANKRD9 (green) and ApoB (purple) in control cells and ANKRD9 overexpressing cells (differentiated) under no treatment, ATP depletion (in ATP depletion medium: no glucose DMEM with 30 uM FCCP and 30 uM oligomycin) for 15 min and ATP depletion for 15 min, then addition of 1 mM ATP for 45 min. The confocal images were taken at 0.5 um under the apical membrane of the cells. Scale bar: 10 um; The data represent three independent experiments. (b) The ATP levels in control and ANKRD9-overexpressing Caco-2 cells were determined by luciferase-based assay. n=3 individual samples per group, please refer to the Source Data File; p-values as indicated by two-way ANOVA with Tukey test; Data are shown as the mean ± SEM.

Supplementary Figure 15

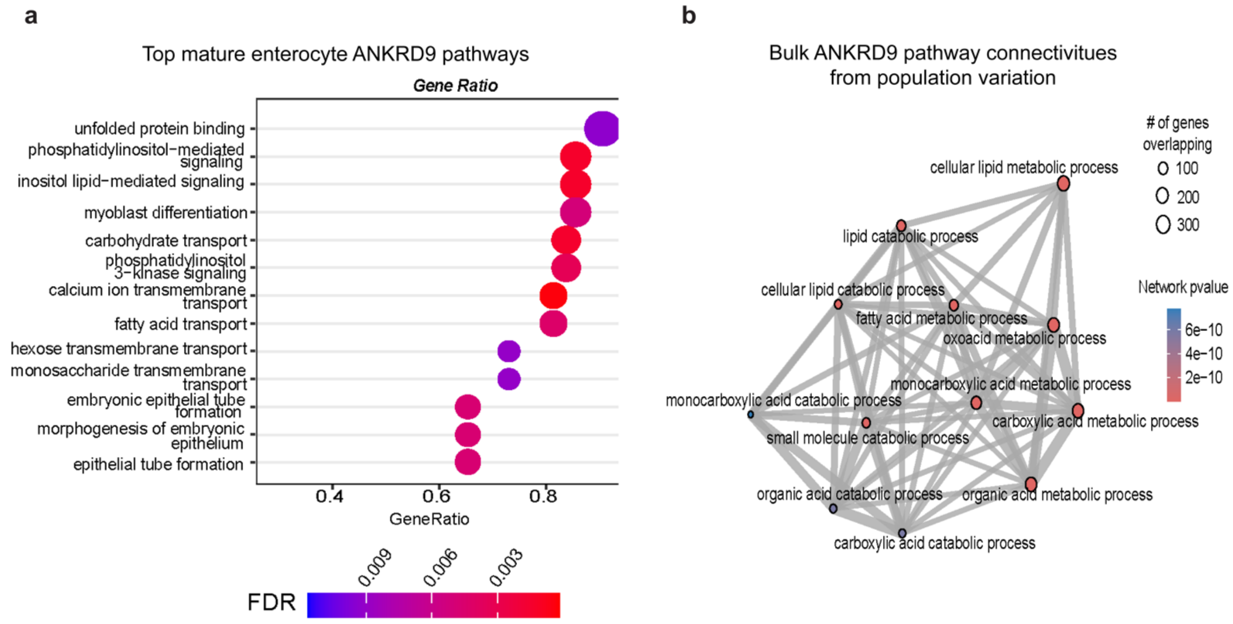

Supplementary Figure 15. Analyses of the top gene-set enrichment GSEA terms arising from ANKRD9-correlated genes

(a) Single-nuclear expression of ANRD9 was correlated with all other genes in mature enterocytes and the GSEA-generated top pathways are shown. (b) Variation in expression of ANKRD9 was correlated with all other genes in small intestine among the 412 individuals available in GTEx. The top pathways are shown as a network graph showing the number of genes overlapping for each pathway. P-values were calculated based on permutation testing standardized for pathway enrichment GSEA analysis and false discovery rate adjustments made based on FDR<0.01.

Supplementary Methods

Protein extraction

Tissues, enteroids, and cell proteins were extracted by homogenizing in lysis buffer (Cell Signaling, #9803) with 1 mM phenylmethanesulfonyl fluoride (PMSF, Sigma, P7626) and 1× Complete Protease Inhibitor Cocktail (Sigma, 11697498001). After centrifugation at 10,000 g for 10 minutes at 4°C, the supernatant was collected as the cell lysate. The protein concentration was then measured using a BCA protein assay, and the sample was stored for the next experiment.

Western blot

Ten to twenty micrograms of extracted protein were resolved on NuPAGE 4-12% Bis-Tris Protein Gels (Thermo Fisher Scientific, NP0323BOX), NuPAGE 3-8% Tris-Acetate Protein Gels (Thermo Fisher Scientific, EA03755BOX), or NativePAGE 4-16% Bis-Tris Gels (Thermo Fisher Scientific, BN1002BOX), and then transferred to PVDF membranes (Bio-Rad, 1620177). The membranes were subsequently incubated with antibodies (antibody list in Supplementary Table 1). Band quantification on Western blots was performed by scanning the blots and determining the band densities using ImageJ software (National Institutes of Health, Bethesda, Maryland, USA).

Lipidomes

Approximately 30 mg tissue were weighed into 2 ml microvials. A lipid extraction solution consisting of 200 ul of 2-propanol with 0.275 ug/ml 13C-labeled lipids was added to the microvials. Two glass beads were added for homogenization using a BeadBeater (15 seconds per cycle, repeated three times). The homogenized samples were then subjected to ultrasonication in ice water for 20 minutes and incubated overnight at −20 °C. On the following day, samples were centrifuged at 18,659 × g and 4 °C for 10 minutes, and the resulting supernatants were transferred to 2 mL glass vials with inserts for UPLC-Q Exactive Orbitrap MS analysis.

A Thermo Vanquish UPLC system coupled with Q-Exactive Orbitrap mass spectrometer equipped with a heated electrospray ionization (HESI) probe (Thermo Fisher Scientific, CA, USA) was used in this study. An Acquity UPLC CSH C18 1.7um 2.1x100mm column (Waters Corp, Milford, MA, USA) was applied for lipid separation in both negative and positive ionization modes with separate injections. Mobile phase A was acetonitrile/H_2O=60/40 (v/v), containing 10 mM ammonium acetate and 0.1% formic acid, and mobile phase B was acetonitrile/2-propanol=10/90 (v/v), containing 10 mM ammonium acetate and 0.1% formic acid. A linear gradient elution program

was set as 30% B from 0-3 min, increasing to 62% B for 2 min and to 82% B for 10 min; after another 1.5 min at 99% B for 1.5 min, the mobile phase composition was then returned to 30% B for 5 min. The total run time was 20 min. The flow rate was 0.35 ml/min and the column temperature was 45 °C. The resolution for data collection in the full scan was 70000 at the ranges of m/z 100-1500. The automatic gain control (AGC) target was 3e6 and the maximum IT was 200 ms. While the dd-MS2/dd-SIM mode parameters were set as a resolution at 17500, AGC target at 1e5, maximum IT of 50ms, loop count at 10, isolation window of 1.2 m/z, and normalized collision energy (NCE) of 20, 50, and 80 eV.

The lipidomes analysis involved the utilization of the MS-DIAL software to analyze all data acquired from the UPLC-QE Orbitrap MS. The raw data acquisition was performed using Xcalibur 4.0 software (Thermo Fisher Scientific, USA). Subsequently, the raw data were converted from the vendor-specific file format (.raw) to the Analysis Base File format (.abf) using the freely available Reifycs ABF converter. After conversion, the MS-DIAL software (version 4.24) was employed for various data processing tasks, including feature detection, spectral deconvolution, peak identification, and alignment between samples. Quality control samples (QC) from each bacteria strain or mice samples were used for peak alignment. During the analysis, specific adducts were selected based on the ionization mode. In positive ionization mode, adducts such as $[M+H]^+$, $[M+NH4]^+$, $[M+Na]^+$, $[M+ACN+H]^+$, $[M+H-H2O]^+$, $[M+H-2H2O]^+$, $[2M+H]^+$, and $[M+2H]^{2+}$ were chosen, while in negative ionization mode, adducts including $[M-H]^-$, [M-H2O-H]-, $[M+Na-2H]^{2-}$, $[M+FA-H]^-$, $[M+Hac-H]^-$, $[2M-H]^-$, and $[M-2H]^{2-}$ were selected. The lipid database settings were kept as default for both positive and negative ion modes. Chemical assignment of molecular features in the samples was performed by comparing the recorded retention time (RT) and m/z information to the reference library constructed from authentic standards. Tolerance windows of 0.05 min for RT and 0.01 Da for m/z were set. To filter the results, a minimal peak count filter of 5,000 or a signal-to-noise ratio (S/N) filter of 10 was applied to all samples. The MS-DIAL analysis generated a comprehensive list of metabolite names, m/z values, RT values, formulas, ontologies, INCHIKEYs, SMILES representations, S/N ratios, and peak areas for high confidence annotations, as well as all unknown molecular features for both positive and negative polarity modes. Specific metabolite features were excluded from the list under the following conditions: (1) if they were detected only in the blank controls, (2) if the coefficient of variation (CV) in the QC samples was higher than 20%, (3) if the annotated compounds were identified in both positive and negative polarity modes and had lower peak areas or S/N ratios, or higher CV values, and (4) if the molecular features were unknown, they were also removed for further analysis[1].

Generation of ANKRD9 Knockout Caco-2 Cells

The guide RNA targeting the human ANKRD9 gene (5'-GGTAATGCGCGTACGCTTGG) was designed using the CRISPOR program to disrupt the third exon of the ANKRD9 gene[2]. It was cloned into the pSpCas9(BB)-2A-GFP plasmid (PX458, a gift from Dr. Feng Zhang, Addgene, #48138) using the following pair of primers (Forward: 5'-CACCGGTAATGCGCGTACGCTTGG; Reverse: 5'-AAACCCAAGCGTACGCGCATTACC) via Golden Gate Assembly[3], followed by Sanger sequencing with primer 5'-GACTATCATATGCTTACCGT to verify the presence of the correct insertion.

The successfully assembled plasmid was then amplified using an endotoxin-free Maxiprep kit (Vazyme, DC202-01) and transfected into Caco-2 cells using the TransIT-LT1 reagent (Mirus Bio, MIR 2304) according to the manufacturer's instructions. Seventy-two hours after transfection, green fluorescent protein-positive cells were sorted by fluorescence-activated cell sorting using a cell sorter (SONY, MA900 Multi-Application) followed by limited dilution to obtain single-cell clones. Genomic DNA from each clone was extracted using the DNeasy Blood & Tissue Kit (Qiagen, 69504), and the fragment containing the Cas9 cut site was amplified using the following pair of primers: Forward: 5'-GCGGGATCCATGCCTTGAG; Reverse: 5'-GAATGCCCACGCGG TTGT. The amplified DNA fragments were then sent for Sanger sequencing, and the results were analyzed using the CRISP-ID[4] program to identify the single-cell-derived clone with *ANKRD9* knockout.

Plasmid construction lentivirus infection

For ANKRD9 overexpressing in HEK293 cells, pLenti6/V5 TOPO-Flag-ANKRD9-mCherry were generated. The N-terminal Flag-tagged full length human ANKRD9 was synthesized by PCR using pcDNA3.1-Flag tagged human ANKRD9 plasmid as a template[5]. Two primers were used for PCR: forward primer (with XbaI site): 5'-GCGTCTAGAATGGATTACAAGGATGACGAC; reverse primer (with BamHI site): 5'-TATGGATCCGCCTTTGCCAGTGAGGTCCAA. PCR products were inserted into pLenti6/V5 TOPO-mCherry (a gift from Dr. Oskar Laur, Addgene, #128062). For ANKRD9 overexpression in Caco2 cells, pFUGW-Flag-ANKRD9-mCherry were generated. The Flag-ANKRD9-mCherry were synthesized by three steps PCR using pLenti6/V5 TOPO-Flag-ANKRD9-mCherry as a template. Flag-ANKRD9-mCherry was inserted into pFUGW plasmid (the original plasmid from Dr. Feng Zhang, Addgene, #52962[6]) by XbaI and EcoRI. To delete 1-63 amino acids of ANKRD9 N-terminus ANKRD9(1-63del), 5'-GCGGCGCGCCCCTTATCGTCGTCATCC and 5'-TGGATGACGACGATAAGGGGCGCGCCGC were used for PCR by Q5® Site-Directed Mutagenesis Kit (New England Biolabs, E0554).

Plasmids sequence was confirmed by Johns Hopkins University School of Medicine Synthesis and Sequencing Facility.

pLenti6/V5 TOPO-Flag-mCherry, pLenti6/V5 TOPO-Flag-ANKRD9-mCherry, pLenti6/V5 TOPO-Flag-ANKRD9(1-63del)-mCherry, pFUGW-Flag-mCherry, pFUGW-Flag-ANKRD9-mCherry or pFUGW-Flag-ANKRD9(1-63del)-mCherry were transferred with pMD2.G (a gift from Dr. Didier Trono, Addgene, #12259) and psPAX2 (a gift from Dr. Didier Trono, Addgene, #12260) into HEK293T cell by lipofectamine 3000 according to the manufacturer's instructions, respectively. After 72 hours transfection, lentiviral particles containing medium was collected, tittered and infected to HEK293 cells. Please refer to individual sections below for the specific treatment condition. Please refer to the plasmid map for detail information in Plasmid map.

pLenti6/V5 TOPO-Flag-mCherry, pLenti6/V5 TOPO-Flag-ANKRD9-mCherry or pLenti6/V5 TOPO-Flag-ANKRD9(1-63del)-mCherry were expressed in HEK293 in DMEM medium (including 10% FBS with 4.5 g glucose/ L) cells by lentivirus infection for 48h for following experiments. Overexpressing of pFUGW-Flag-mCherry, pFUGW-Flag-ANKRD9-mCherry or pFUGW-Flag-ANKRD9(1-63del)-mCherry in Caco2 cells by lentivirus infection for 72h. The mCherry positive cells we sorted by cell sorter (SONY-MA900 Cell Sorter), to generate stable cell lines (ANKRD9 negtive cells, ANKRD9 low expressing cells, ANKRD9 high expressing cells, ANKRD9(1-63del) low expressing cells and ANKRD9(1-63del) high expressing cells) for next experiments.

Isolation of ANKRD9-mCherry complexes

ANKRD9-mCherry complexes were obtained by fractionation of HEK293 cells; cells transfected with Flag-mCherry were used as a control. All the experiments were performed at 4°C. About $1x10^6$ cells grown in 10 cm^2 dish were washed once by cold PBS and collected in 1ml SMT buffer (including 0.27M sucrose and 10 mM MOPS-Tris (pH6.8) as described previously[7]. Cells were homogenized by 15 strokes through a 28G needle. Then the cell homogenates were separated (Suppl-Fig. 8A) by step-1: centrifugation at 500 × g for 5 minutes, the supernatant was transferred to a new tube, the pellets were washed twice by SMT buffer, added lysis buffer (Cell signaling, #9803) with 1mM PMSF (Sigma, P7626) and 1 x Complete Protease Inhibitor cocktail (Sigma, 11697498001) and centrifuged at 18000 × g for 10 minutes, saved the supernatant was fraction 1. Step-2: the supernatant (from step-1) was centrifuged at 3000 × g for 5 minutes, the supernatant was transferred to a new tube, the pellets were processed as step-1 and saved the supernatant was fraction 2. Step-3: the supernatant (from step-2) was centrifuged at 18000 × g for 30 minutes, the supernatant was fraction 4, the pellets were processed as step-1 and saved

the supernatant was fraction 3. Protein adjustment in all four fractions, added blue native sample buffer for blue native western blot assay and for standard western blot assay.

Human single-nuclear sequencing data and analysis

Human single-nuclear sequencing analyses – Intestine single-nuclear RNA-Seq data was acquired from FigShare: https://figshare.com/articles/dataset/Tabula_Sapiens_v2/27921984, then clustered using Seurat and labelled using the "free annotations" slot. A filtered and normalized counts matrix was then used to analyze expression patterns. ANKRD9 expression was correlated to other genes using WGCNA::Bicor() function and corresponding pathway enrichments calculated using gseGO package where the correlation coefficient (bicor) was used as the weight and all genes detected in the single-cell seq experiment used as background. Human population variation of ANKRD9 – Bulk expression of ANKRD9 was analyzed from GTEx v10[8]. Specifically, ANKRD9 expression was correlated with all other genes in small intestine and resulting using the WGCNA::Bicor() function. Pathway enrichments from resulting genetic correlations were calculated using gseGO() and correlation coefficient (bicor) was used as the weight with all genes detected in the intestine used as background. The custom code generated for this study was uploaded to GitHub: https://github.com/mingqizh/ANKRD9-Intestine-Integration/tree/main.

Plasmids map

pLenti6/V5 TOPO-Flag-mCherry

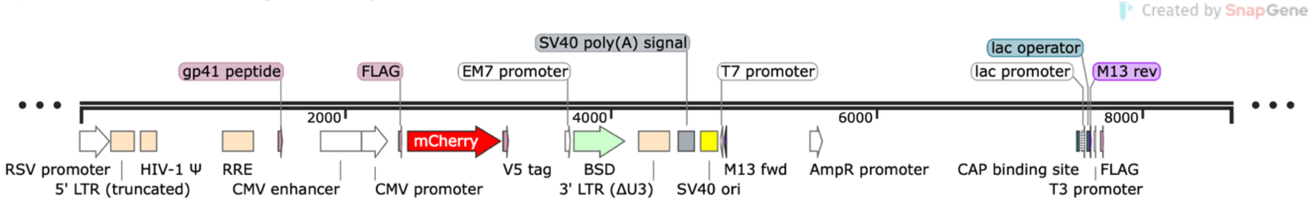

pLenti6/V5 TOPO-Flag-ANKRD9-mCherry or pLenti6/V5 TOPO-Flag-ANKRD9(1-63del)-mCherry

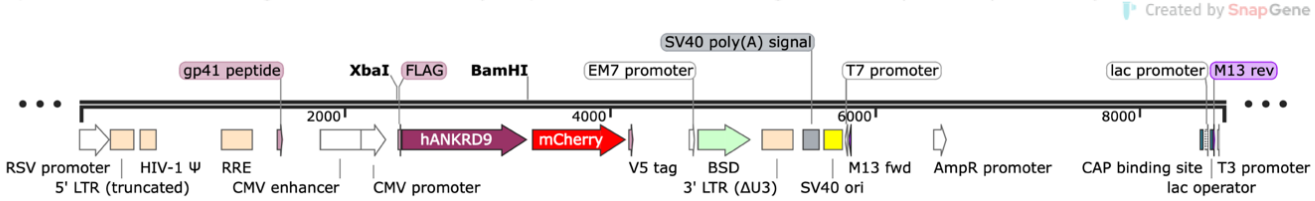

pFUGW-Flag-mCherry

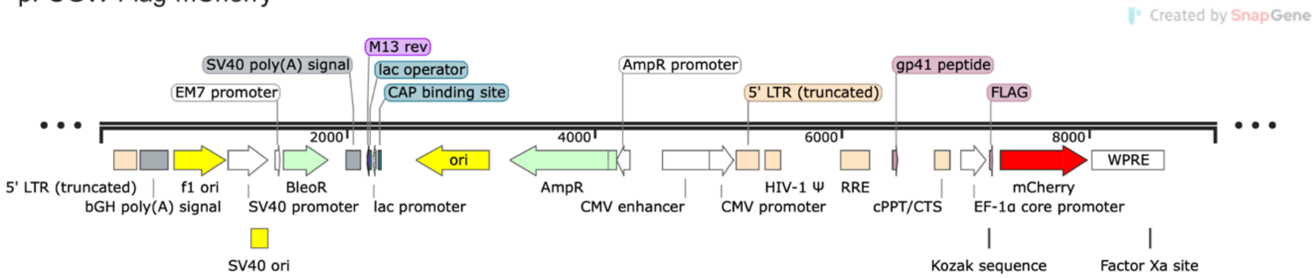

pFUGW-Flag-ANKRD9-mCherry or pFUGW-Flag-ANKRD9(1-63del)-mCherry

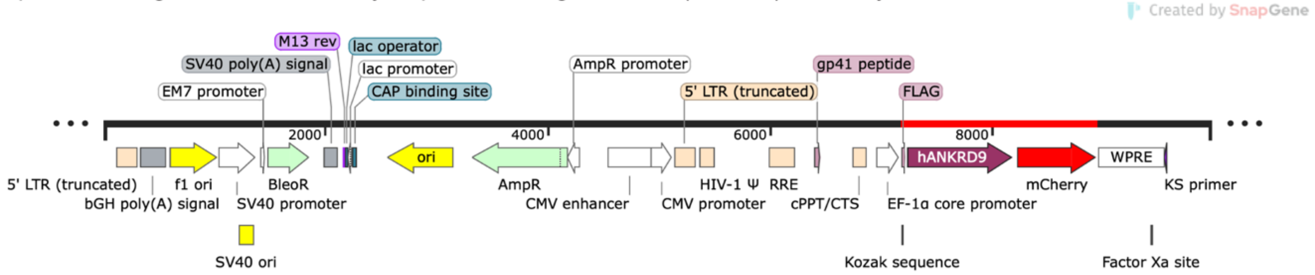

Supplementary Table 1: Antibody List

Antibody	Catalog Number and Lot Number	Concentration
Rabbit anti-Lac Z (beta galactosidase)	Thermo Fisher Scientific, A11132, Lot: 2181017	WB: 1/5000
Mouse anti-GM130	BD Biosciences, 610823, Lot: 3200321	WB: 1/1000; IHC: 1/100
Rabbit anti-IMPDH2	Abcam, ab131158, Lot: 1003869	WB: 1/1000; IHC: 1/200
Rabbit anti-GAPDH	Cell signaling, #5174	WB: 1/1000
Rabbit anti-mCherry	Abcam, ab183628	WB: 1/1000
Sheep anti-TGN46	GeneTex, GTX74290, Lot: 822401871	WB: 1/1000, IHC: 1/150
Rabbit anti-ANKRD9	Sigma, SAB2108422, Lot: QC25610	WB: 1/1000; IHC: 1/75
Mouse anti-Flag	Sigma, F1804	WB: 1/1000
Rabbit anti-β-actin	Cell Signaling, #4967	WB: 1/1000
Mouse anti-α-tubulin	Sigma, T8203	WB: 1/1000
Rabbit anti-PRPS1	Proteintech, 15549-1-AP, Lot: 00096546&00149635	WB: 1/1000; IHC: 1/75
Goat anti-ApoB	Abcam, ab7616, Lot: 1029643-1	WB: 1/2000, IHC:1/75

Reference:

1. Chen, L. *et al.* Mapping pesticide-induced metabolic alterations in human gut bacteria. *Nat Commun* **16**, 4355 (2025).
2. Concordet, J.P. & Haeussler, M. CRISPOR: intuitive guide selection for CRISPR/Cas9 genome editing experiments and screens. *Nucleic Acids Res* **46**, W242-W245 (2018).
3. Ran, F.A. *et al.* Genome engineering using the CRISPR-Cas9 system. *Nat Protoc* **8**, 2281-2308 (2013).
4. Dehairs, J., Talebi, A., Cherifi, Y. & Swinnen, J.V. CRISP-ID: decoding CRISPR mediated indels by Sanger sequencing. *Sci Rep* **6**, 28973 (2016).
5. Hayward, D. *et al.* ANKRD9 is a metabolically-controlled regulator of IMPDH2 abundance and macro-assembly. *J Biol Chem* **294**, 14454-14466 (2019).
6. Sanjana, N.E., Shalem, O. & Zhang, F. Improved vectors and genome-wide libraries for CRISPR screening. *Nat Methods* **11**, 783-784 (2014).
7. Brunner, Y. *et al.* Proteomics analysis of insulin secretory granules. *Mol Cell Proteomics* **6**, 1007-1017 (2007).
8. Consortium, G.T. The GTEx Consortium atlas of genetic regulatory effects across human tissues. *Science* **369**, 1318-1330 (2020).
