# Supplementary material for: Enterocytes rely on purine biosynthesis/salvage pathway to facilitate dietary fat absorption
Source: Nat Commun. 2026 Mar 13;17:3888. doi: 10.1038/s41467-026-70332-3 (PMC13125253; doi:10.1038/s41467-026-70332-3)
Supplement: Supplementary file 2 — Reporting Summary [file 41467_2026_70332_MOESM2_ESM.pdf]

Corresponding author(s): Svetlana Lutsenko and Yu Wang

Last updated by author(s): January 12, 2026

Reporting Summary

Nature Portfolio wishes to improve the reproducibility of the work that we publish. This form provides structure for consistency and transparency in reporting. For further information on Nature Portfolio policies, see our Editorial Policies and the Editorial Policy Checklist.

Statistics

For all statistical analyses, confirm that the following items are present in the figure legend, table legend, main text, or Methods section.

n/a	Confirmed	
☐	☒	The exact sample size (*n*) for each experimental group/condition, given as a discrete number and unit of measurement
☐	☒	A statement on whether measurements were taken from distinct samples or whether the same sample was measured repeatedly
☐	☒	The statistical test(s) used AND whether they are one- or two-sided<br>*Only common tests should be described solely by name; describe more complex techniques in the Methods section.*
☐	☒	A description of all covariates tested
☐	☒	A description of any assumptions or corrections, such as tests of normality and adjustment for multiple comparisons
☐	☒	A full description of the statistical parameters including central tendency (e.g. means) or other basic estimates (e.g. regression coefficient) AND variation (e.g. standard deviation) or associated estimates of uncertainty (e.g. confidence intervals)
☐	☒	For null hypothesis testing, the test statistic (e.g. *F*, *t*, *r*) with confidence intervals, effect sizes, degrees of freedom and *P* value noted<br>*Give P values as exact values whenever suitable.*
☒	☐	For Bayesian analysis, information on the choice of priors and Markov chain Monte Carlo settings
☒	☐	For hierarchical and complex designs, identification of the appropriate level for tests and full reporting of outcomes
☒	☐	Estimates of effect sizes (e.g. Cohen's *d*, Pearson's *r*), indicating how they were calculated

Our web collection on statistics for biologists contains articles on many of the points above.

Software and code

Policy information about availability of computer code

Data collection	Confocal data was acquired in Zen Blue V3.2(Zeiss) software.
Data analysis	Microsoft Excel for Microsoft 365, Graphpad Prism 10, Cellprofiler, Imairs, Zeiss Zen software, and Image J.

For manuscripts utilizing custom algorithms or software that are central to the research but not yet described in published literature, software must be made available to editors and reviewers. We strongly encourage code deposition in a community repository (e.g. GitHub). See the Nature Portfolio guidelines for submitting code & software for further information.

Data

Policy information about availability of data

All manuscripts must include a data availability statement. This statement should provide the following information, where applicable:

- Accession codes, unique identifiers, or web links for publicly available datasets
- A description of any restrictions on data availability
- For clinical datasets or third party data, please ensure that the statement adheres to our policy

All data supporting the findings of this study are provided in the manuscript, supplementary information file, and Source Data files. The data of proteomics, lipidomics, metabolomics and nucleotide measurements are deposited to the public repository (please refer to the Data Availability section in the manuscript). The generated codes for this study has been uploaded to GitHub (please refer to the Code Availability section in the manuscript). The Source Data File accompanying this publication includes data from individual experiments utilized in deriving the final figures. The Source Data File accompanying this manuscript.

Research involving human participants, their data, or biological material

Policy information about studies with human participants or human data. See also policy information about sex, gender (identity/presentation), and sexual orientation and race, ethnicity and racism.

Reporting on sex and gender	Human jejunal enteroids were provided by Dr. Foulke-Abel from Johns Hopkins University School of Medicine.
Reporting on race, ethnicity, or other socially relevant groupings	N/A
Population characteristics	N/A
Recruitment	N/A
Ethics oversight	N/A

Note that full information on the approval of the study protocol must also be provided in the manuscript.

Field-specific reporting

Please select the one below that is the best fit for your research. If you are not sure, read the appropriate sections before making your selection.

☒ Life sciences ☐ Behavioural & social sciences ☐ Ecological, evolutionary & environmental sciences

For a reference copy of the document with all sections, see nature.com/documents/nr-reporting-summary-flat.pdf

Life sciences study design

All studies must disclose on these points even when the disclosure is negative.

Sample size	We did not precalculate the sample size ahead. Refer to previous studies, and optimized the significant difference, we used at least three biological replicates were achieved for all the experiment. Such as sample size are typical for ex vivo experiments. for in vivo experiment, a sample size >3 mice were used per experimental group. The specific numbers are provided in the figure legends and the Source Data file.
Data exclusions	No data was excluded from the analysis.
Replication	All the attempts at replicating the results were successful. Each experiment was successfully replicated at least three independent times.
Randomization	For animal experiment, the animal from the same genotype were allocated to various groups(0 min, 15 min, 30 min, 60min, 90min and 120 min) randomly.
Blinding	Blinding was performed when possible, for example for quantification of the number of Prps1 vesicles per cell, quantification of the lipid are per cell, and quantification of Golgi size from EM images, nucleotides measurement by LC-MS, insulin tolerance test, or blood glucose measurement.

Reporting for specific materials, systems and methods

We require information from authors about some types of materials, experimental systems and methods used in many studies. Here, indicate whether each material, system or method listed is relevant to your study. If you are not sure if a list item applies to your research, read the appropriate section before selecting a response.

Materials & experimental systems

n/a	Involved in the study	
☐	☒	Antibodies
☐	☒	Eukaryotic cell lines
☒	☐	Palaeontology and archaeology
☐	☒	Animals and other organisms
☒	☐	Clinical data
☒	☐	Dual use research of concern
☒	☐	Plants

Methods

n/a	Involved in the study	
☒	☐	ChIP-seq
☒	☐	Flow cytometry
☒	☐	MRI-based neuroimaging

Antibodies

Antibodies used	Rabbit anti-Lac Z (beta galactosidase) Thermo Fisher, A11132, Lot: 2181017, WB: 1/5000<br>Mouse anti-GM130 BD Biosciences, 610823, Lot: 3200321, WB: 1/1000; IHC: 1/100<br>Rabbit anti-IMPDH2 Abcam, ab131158, Lot: 10033869, WB: 1/1000; IHC: 1/200<br>Rabbit anti-GAPDH Cell signaling, #5174 WB: 1/1000<br>Rabbit anti-mCherry Abcam, ab183628 WB: 1/1000<br>Sheep anti-TGN46 GeneTex, GTX74290, Lot: 822401871, IHC: 1/150,WB: 1/1000<br>Rabbit anti-ANKRD9 Sigma, SAB2108422 , Lot: QC25610, WB: 1/1000; IHC: 1/75<br>Mouse anti-Flag Sigma, F1804, WB: 1/1000<br>Rabbit anti-beta-actin Cell Signaling, #4967 WB: 1/1000<br>Mouse anti-alpha-tubulin Sigma, T8203 WB: 1/1000<br>Rabbit anti-PRPS1 Proteintech, 15549-1-AP, Lot: 00096546&00149635, WB: 1/1000; IHC: 1/75<br>Goat anti-ApoB Abcam, ab7616, Lot: 1029643-1, WB: 1/2000, IHC:1/75
Validation	Validation statements for all used the antibodies are available at the websites of the commercial provider.<br>https://www.thermofisher.com/antibody/product/beta-Galactosidase-Antibody-Polyclonal/A-11132<br>https://www.bdbiosciences.com/en-us/products/reagents/microscopy-imaging-reagents/immunofluorescence-reagents/purified-mouse-anti-gm130.610823?tab=product_details<br>https://www.abcam.com/en-us/products/primary-antibodies/impdh2-antibody-epr8364b-ab131158<br>https://www.cellsignal.com/products/primary-antibodies/gapdh-d16h11-xp-rabbit-mab/5174<br>https://www.abcam.com/en-us/products/primary-antibodies/mcherry-antibody-ab183628<br>https://www.genetex.com/Product/Detail/TGN46-antibody/GTX74290<br>https://www.sigmaaldrich.com/US/en/product/sigma/sab2108422<br>https://www.sigmaaldrich.com/US/en/product/sigma/f1804<br>https://www.cellsignal.com/products/primary-antibodies/b-actin-antibody/4967<br>https://www.sigmaaldrich.com/US/en/product/sigma/t8203<br>https://www.ptglab.com/Products/PRPS1-Antibody-15549-1-AP.htm<br>https://www.abcam.com/en-us/products/primary-antibodies/apolipoprotein-b-antibody-ab7616

Eukaryotic cell lines

Policy information about cell lines and Sex and Gender in Research

Cell line source(s)	Caco-2 cells were obtained from Dr. Mark Donowitz from Johns Hopkins University School of Medicine (Male). HEK293T cells were purchased from ATCC(CRL-3216, female). Mouse enteroid were isolated from 14 week old male mice.
Authentication	We did not authenticate the cell lines of Caco2 and HEK293T, the genotypes of Ankrd9+/+ (wild type)and Ankrd9-/- (knockout) were conformed by PCR-based genotyping.
Mycoplasma contamination	The cell line tested negative for mycoplasma.
Commonly misidentified lines<br>(See ICLAC register)	No commonly misidentified cell line were used.

Animals and other research organisms

Policy information about studies involving animals; ARRIVE guidelines recommended for reporting animal research, and Sex and Gender in Research

Laboratory animals	Ankrd9-/- mice on C57BL/6N-Atm1Brd background were obtained from the Mutant Mouse Resource and Research Center (MMRRC) at the University of California at Davis (UC Davis, RRID: MMRRC_046599-UCD, C57BL/6N-Atm1BrdAnkrd9-tm1/(KOMP)Wsti/Mmucd). In these mice , exon 3 of Ankrd9 was replaced by FRT- LacZ - loxp - neomycin resistance (Neo) cassette; this resulted in the loss of Ankrd9 expression and the expression of LacZ under the endogenous Ankrd9 promoter. Ankrd9-/- and Ankrd9+/+ littermate controls were generated by heterozygous Ankrd9+/- breeding and were maintained on C57BL/6N background.
Wild animals	N/A
Reporting on sex	Based on the body fat mass analysis, there is no difference between female mice (Ankrd9-/-) and wild-type (Ankrd9+/+), thus fourteen weeks old homozygous Ankrd9 knockout male mice (Ankrd9-/-) and wild-type (Ankrd9+/+) male littermates were used in the experiments.
Field-collected samples	N/A
Ethics oversight	All animal protocols were approved by the Institutional Animal Care and Use Committee of the Johns Hopkins University (JHU ACUC, protocol #M017M385, MO20M333 and MO23M337). The mice were maintained under specofoc pathogen-free condition with a controlled temperature of 22-24oC, relatived humidity of 40-60% and a 14-h light/10-h dark cycle with free access to water and food unless indicated otherwise.

Note that full information on the approval of the study protocol must also be provided in the manuscript.

Plants

Seed stocks	Not applicable for this study
Novel plant genotypes	Not applicable for this study
Authentication	Not applicable for this study
